# Regorafenib Induced Interstitial Pneumonia in a Patient With Refractory Rectal Cancer

**DOI:** 10.1002/rcr2.70286

**Published:** 2025-07-30

**Authors:** Tomo Tsunoda, Yoshiyuki Oyama, Ryota Miyamoto, Taisuke Ito, Takuro Akashi, Kazuo Tsuchiya, Masaki Ikeda

**Affiliations:** ^1^ Department of Respiratory Medicine Shizuoka Saiseikai General Hospital Shizuoka Japan

**Keywords:** colorectal cancer, drug‐induced interstitial lung disease, interstitial pneumonia, multi‐targeted tyrosine kinase inhibitor, regorafenib

## Abstract

Regorafenib, a multi‐targeted tyrosine kinase inhibitor (TKI), is indicated for refractory colorectal carcinoma, gastrointestinal stromal tumours (GIST), and hepatocellular carcinoma (HCC). We present a case involving a 66‐year‐old male patient with refractory colorectal cancer who developed interstitial pneumonia as a consequence of regorafenib therapy. Three months following the initiation of regorafenib administration, a chest computed tomography scan revealed bilateral ground‐glass opacities, a characteristic finding in interstitial lung disease. This case illustrates a relatively rapid progression of regorafenib‐induced interstitial lung disease following its radiographic manifestation. Clinicians should remain vigilant for this potential pulmonary toxicity in patients receiving regorafenib, even with an apparently short latency period after treatment commencement. Early recognition and prompt intervention are crucial in managing this adverse event.

## Graphical abstract

We present a case involving a 66‐year‐old male patient with refractory colorectal cancer who developed interstitial pneumonia as a consequence of regorafenib therapy. This case illustrates a relatively rapid progression of regorafenib‐induced interstitial lung disease following its radiographic manifestation. Early recognition and prompt intervention are crucial in managing this adverse event.

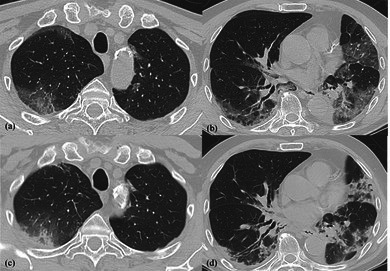

## 1 Introduction

Regorafenib, a multi‐targeted TKI, inhibits tumour cell proliferation and angiogenesis by targeting key receptors, including vascular endothelial growth factor receptors (VEGFR) 1–3, platelet‐derived growth factor receptors (PDGFR) α/β, fibroblast growth factor receptor 1 (FGFR) 1, KIT, RET and TIE2 [1]. Approved in Japan for unresectable or refractory colorectal cancer, refractory GIST, and refractory HCC [2, 3, 4], its Phase 3 trials showed no drug‐related interstitial lung disease (ILD). However, post‐marketing surveillance in Japan has revealed rare cases [5]. With limited understanding and only one prior report of exacerbated pre‐existing interstitial pneumonia following lenvatinib after regorafenib [6], the clinical course of isolated regorafenib‐induced ILD remains unclear. We present a case of interstitial pneumonia likely due to regorafenib monotherapy.

## 2 Case Report

**FIGURE 1:**
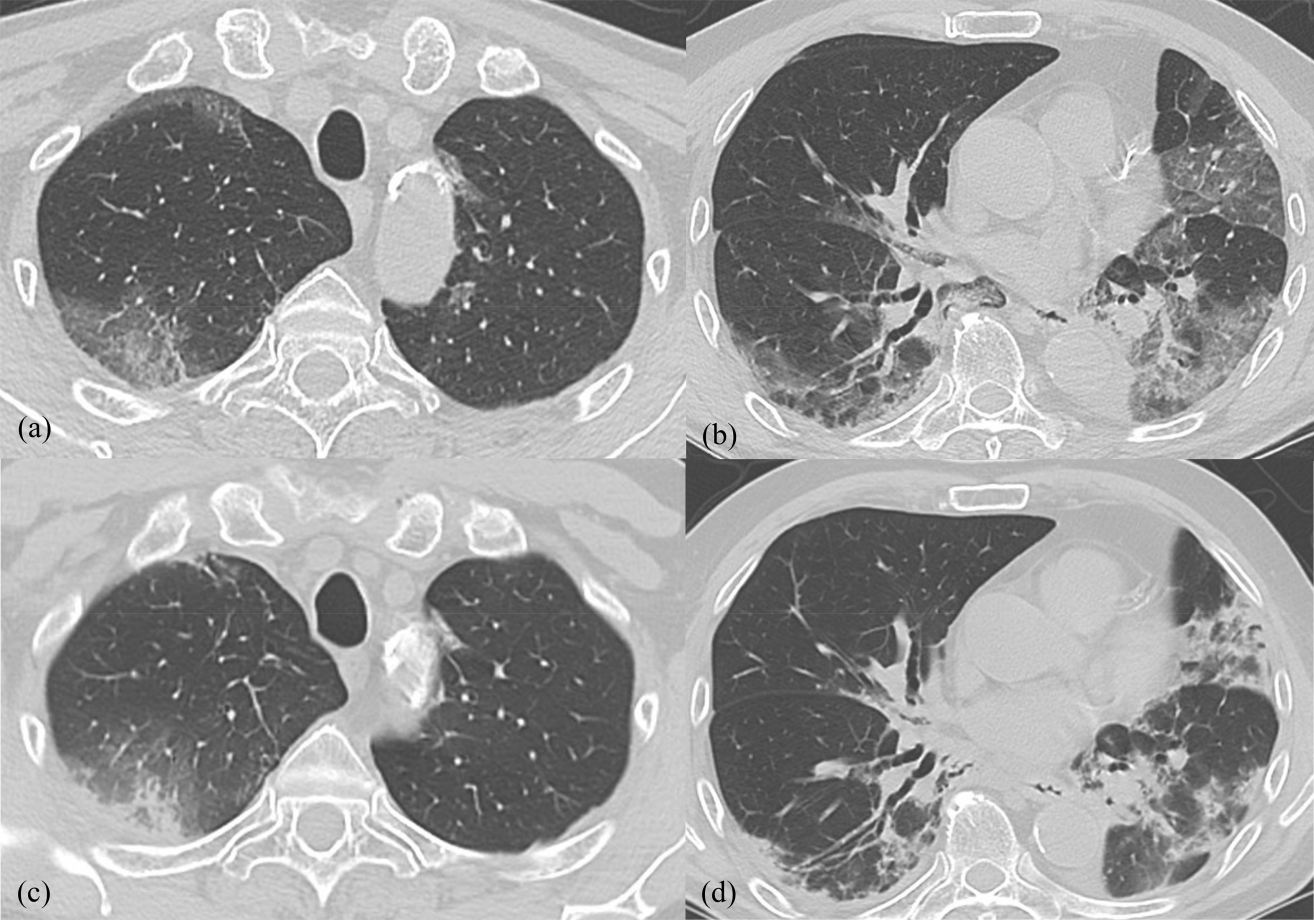
(a, b) High‐resolution chest tomography (HRCT) image at 3 months after starting regorafenib. Ground‐glass opacity (GGO) appeared in both lung fields. (c, d) HRCT image at 1 week after GGO appearance. Patchy infiltration shadow and consolidation replaced GGO in both lung fields.

Diagnosed with Stage IIIb rectal cancer in August 2020, a 66‐year‐old man underwent low anterior resection, followed by 6 months of adjuvant therapy with capecitabine and oxaliplatin. Subsequently, a left pulmonary metastasis necessitated left lower lobectomy in June 2021. January 2023 revealed a right lung metastasis, initially managed with irinotecan and bevacizumab, then continued with irinotecan alone. July 2023 brought detection of new pulmonary and mediastinal metastases, treated with radiation and 6 months of tegafur/gimeracil/oteracil. A left upper lobe lesion was identified in January 2024, leading to 7 months of trifluridine/tipiracil/hydrochloride/bevacizumab. Ultimately, a recurrent right upper lobe metastasis prompted the start of regorafenib at 160 mg/day in September 2024. Three months following the initiation of regorafenib therapy, the patient presented with cephalalgia in January 2025 (110 days after regorafenib initiation). A cranial CT scan revealed cerebral metastases, necessitating regorafenib discontinuation (113 days after regorafenib initiation) and subsequent craniotomy (7 days after regorafenib discontinuation). Post‐craniotomy thoracic radiography and CT demonstrated non‐regional ground‐glass opacities (127 days after regorafenib initiation) (Figure 1). Empirical antibiotic therapy yielded no clinical improvement. Serological and physical examinations excluded opportunistic infections, non‐tuberculous mycobacterial infection and collagen vascular diseases. No history of significant inhaled antigen exposure was elicited. Regorafenib‐induced ILD, graded as Grade 3 according to the Common Terminology Criteria for Adverse Events (CTCAE) version 5.0, was suspected.

Upon admission, the patient reported dyspnea on exertion (Modified British Medical Research Council [mMRC] grade 2). Initial vital signs were: blood pressure 129/69 mmHg, temperature 37.3°C and peripheral oxygen saturation (SpO2) of 97% on 2 L per minute of nasal cannula oxygen. Initial laboratory investigations revealed elevated Krebs von den Lungen‐6 (KL‐6) at 578 U/mL (reference range < 500 U/mL) and C‐reactive protein (CRP) at 9.684 mg/dL (Table 1).

**TABLE 1:** The serum test results at the time of admission to our department.

Haematology			Biochemistry			Serology		
WBC	6140	/μL	Na	138	mEq/L	CRP	9.684	mg/dL
Neu	73.7	%	K	5.1	mEq/L	RF	14.1	IU/mL
Lym	15.1	%	Cl	99	mEq/L	ANA (speckles pattern)	40	Times
Mon	9.0	%	TP	6.1	g/dL	Anti‐CCP antibody	2.12	ng/mL
Eos	2.0	%	Alb	2.8	g/dL	Anti‐RNP antibody	< 2.0	U/mL
Bas	0.2	%	AST	31	U/L	Anti‐SM antibody	< 1.0	U/mL
RBC	378	×10^4^/μL	ALT	41	U/L	Anti‐SS‐A antibody	< 1.0	U/mL
Hb	13.0	g/dL	LDH	310	U/L	Anti‐SS‐B antibody	< 1.0	U/mL
Plt	31.3	×10^4^/μL	BUN	10	mg/dL	Anti‐Scl‐70 antibody	< 1.0	U/mL
Infection			Cre	0.77	mg/dL	Anti‐ARS antibody	(−)	
CMV PCR	0	IU/mL	eGFR	77.6	mL/min/1.73m^2^	Anti‐Jo‐1 antibody	< 1.0	U/mL
*β*‐d glucan	8.5	pg/mL	Glu	124	mg/dL	Anti‐centromere antibody	< 2.0	U/mL
*Aspergillus* antigen	0.1	(−)	HbA1c	6.6	%	Anti‐RNP III antibody	< 5	
*Aspergillus* IgG antibody	1.4	(−)	KL‐6	578	U/mL	PR3‐ANCA	< 1.0	IU/mL
T‐SPOT	(−)		SP‐D	82.5	ng/mL	MPO‐ANCA	< 1.0	IU/mL

Abbreviations: Alb, albumin; ALT, alanine aminotransferase; ANA, antinuclear antibody; Anti‐ARS antibody, Anti‐Aminoacyl‐tRNA Synthetases antibody; Anti‐RNP III antibody, anti‐RNA polymerase III antibody; Anti‐Scl‐70 antibody, anti‐Scleroderma‐70 antibody; anti‐SS‐A antibody, anti‐Sjögren's‐syndrome‐related antigen A autoantibodies; anti‐SS‐B antibody, anti‐Sjögren's‐syndrome‐related antigen B autoantibodies; AST, aspartate aminotransferase; Bas, basophil; BUN, blood urea nitrogen; CMV PCR, cytomegalovirus PCR; Cre, creatinine; CRP, C‐reactive protein; eGFR, estimated glomerular filtration rate; Eos, eosinophil; Glu, glucose; Hb, haemoglobin; HbA1c, glycated haemoglobin; KL‐6, krebs von den luen‐6; LDH, lactate dehydrogenase; Lym, lymphocyte; Mon, monocyte; MPO‐ANCA, myeroper oxidase antineutrophil cytoplasmic antibody; Neut, neutrophil; Plt, platelet; PR3 ANCA, proteinase3 antineutrophil cytoplasmic antibody; RBC, red blood cell; RF, rheumatoid factor; SP‐D, surfactant protein D; TP, total protein; T‐SPOT, T‐SPOT‐TB; WBC, white blood cell.

**FIGURE 2:**
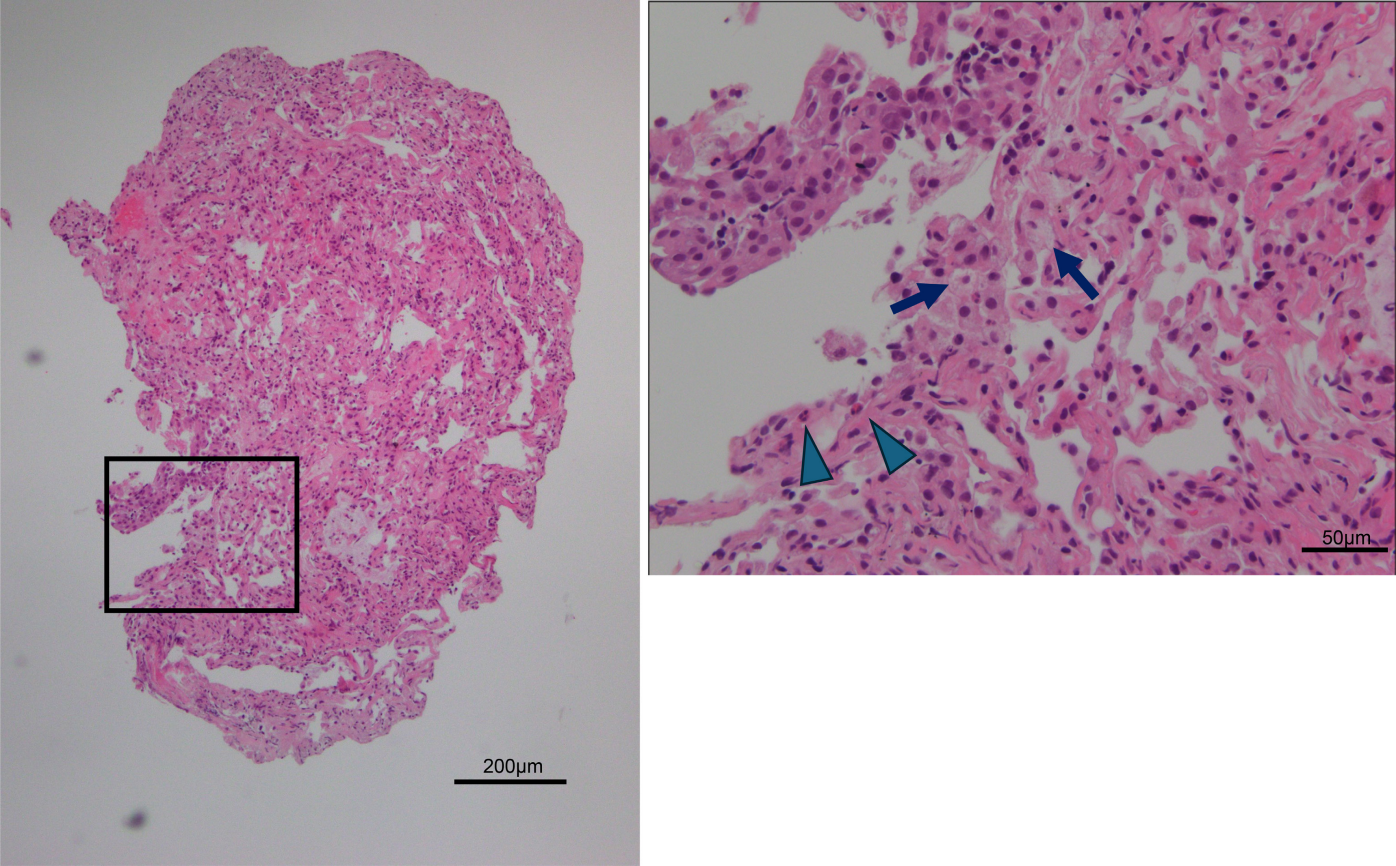
Histological findings of the transbronchial lung biopsy specimen. A histological examination was performed with Haematoxylin and Eosin staining. The square area of the low‐magnification photomicrograph is shown as a high‐magnification photomicrograph. The arrows indicate the infiltration of foamy macrophages and the arrowheads indicate the infiltration of eosinophils into the alveolar interstitium. A scale bar is shown in each figure.

**FIGURE 3:**
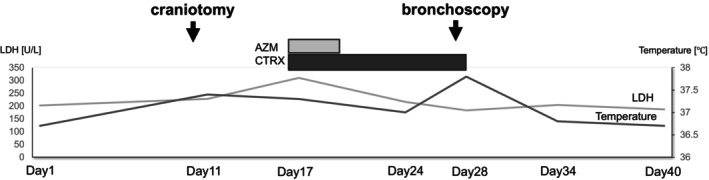
Clinical course of the patient after admission. AZM, Azithromycin; CTRX, ceftriaxone; LDH, lactate dehydrogenase.

Bronchoscopy with bronchoalveolar lavage (BAL) of the right middle lobe revealed a total cell count of 2.2 × 10^5^/mL, predominantly histiocytes (85.4%); no malignant cells were seen (Table 2). Transbronchial lung biopsy (TBLB) from the right upper lobe showed mild alveolar wall thickening and interstitial infiltration of eosinophils and foamy macrophages, without Masson bodies (Figure 2). Based on these findings, regorafenib‐induced interstitial pneumonia was diagnosed. Due to the absence of fever or respiratory failure, the patient was monitored without immunosuppression, and pulmonary opacities resolved spontaneously (Figure 3).

**TABLE 2:** The results of bronchoalveolar lavage fluid (BALF).

Total cell counts	2.2 × 10^5^	/mL
Cell fractionation		
Alveolar macrophages	85.4	%
Lymphocytes	6.6	%
Neutrophils	7.6	%
Eosinophils	0.4	%
Basophils	0.0	%
CD4/8 ratio^a^	0.62	
Cytology	Class I	
BALF culture	Negative	

^a^ CD4^+^/CD8^+^T‐lymphocyte ratio.

## 3 Discussion

Regorafenib has a structure similar to sorafenib but with a fluorine substitution. This modification leads to enhanced inhibition of angiogenesis‐related receptors, particularly VEGFR2 and FGFR1, as well as other kinases (VEGFR1, VEGFR3, RAF, TIE2, KIT, RET, BRAF). While drug‐induced lung injury is a recognised adverse effect of sorafenib [7, 8, 9, 10], interstitial pneumonia as an isolated event is rarely reported with regorafenib.

Initial CTCAE Grade classification due to minimal respiratory compromise, low‐grade fever and oxygen needs hinted at Grade 3 pneumonitis. However, the patient remained stable and improved without corticosteroids, leading to a conservative management decision.

While the exact mechanism of regorafenib‐induced ILD remains unclear, its pulmonary toxicity likely stems from the inhibition of the VEGF signalling pathway, a key target in its antitumor activity. VEGF is crucial for maintaining normal lung tissue homeostasis, including endothelial cell survival and permeability regulation [11]. Consequently, VEGF inhibition by regorafenib can disrupt these processes in healthy lung tissue. This is supported by the observation of pulmonary toxicity with other VEGF inhibitors like bevacizumab (Monoclonal antibody) and sunitinib (TKI) [12, 13]. However, the contribution of inhibiting other kinases targeted by regorafenib (e.g., FGFR, PDGFR, KIT, RET, BRAF) to this pulmonary toxicity cannot be excluded [1].

Radiological findings of regorafenib‐induced interstitial pneumonia are not well‐established. A prior report noted ground‐glass opacities (GGO) on HRCT [14], consistent with our initial findings. However, the HRCT pattern evolved to patchy alveolar opacities resembling organising pneumonia with fibrosis. The subsequent resolution of these abnormalities after regorafenib discontinuation suggests that progressive fibrosis did not occur and that earlier cessation might prevent residual lung damage.

Post‐marketing data in refractory HCC showed a 0.5% incidence (5/910) of interstitial pneumonia, and 0.67% (23/3383) in refractory colorectal cancer or GIST. Ethnic variability may explain the observed DILD incidence. Japanese individuals might be more susceptible to drug‐induced ILD, though regorafenib‐specific data are lacking [15]. Lenvatinib, pazopanib, sorafenib, and regorafenib were compared as multi‐targeted TKIs sharing VEGFR and other kinase inhibitory effects (Table 3). While no direct correlation between treatment duration and outcome was observed, sorafenib showed a high mortality (4/6 cases) despite drug withdrawal and steroids.

**TABLE 3:** Summary of the patient with multi‐kinase inhibitor‐induced interstitial pneumonia reported in the literature.

	Case of study, |reference|	Age	Sex	Cancer type	PS	Smoking history	Time from first administration to the onset of drug‐induced interstitial pneumonitis (month)	Pattern of HRCT	KL‐6 (U/mL)	Lymphocyte ratio of BAL (%)	TBLB	DLST	Treatment	Outcome
Lenvatinib	Case Res Oncol 11: 75–80, 2018 [16]	67	M	Cancer of unknown primary	0	Former	1	Bilateral GGO	582	61.10%	No data	(+)	Discontinuation	Recovered
	Clin J Gastroenterol 12: 355–360, 2019 [17]	59	M	HCC	0	Former	1	Bilateral GGO	1283	No data	No data	(−)	Discontinuation mPSL1000 mg	Recovered
	Intern Med 61: 1211–1217, 2022 [18]	84	M	HCC	1	Former	4	Bilateral GGO → Bilateral infiltration shadow	1906	20.80%	Infiltration of lymphocyte into alveolar interstitium	(+)	Discontinuation, mPSL500 mg	Recovered
Pazopanib	Respiratory Medicine Case Reports 30 (2020) 10112 [19]	73	M	Leiomyosarcoma	No data	No data	4	Bilateral GGO	1686	No data	Organising pneumonitis, Masson body	No data	Discontinuation, PSL0.5 mg/kg	Recovered
	BMJ Case Rep 2020; 13:e235177 [20]	75	M	RCC	No data	Never	3	Bilateral GGO	364	No data	No data	(+)	Discontinuation, mPSL1000 mg	Recovered
	Inter Med 56: 79–83, 2017 [21]	74	M	Leiomyosarcoma	2	Current	2	Bilateral GGO, traction bronchiectasis	428	No data	No data	No data	Discontinuation, steroid	Recovered
Sorafenib	Lung Cancer 67 (2010) 248–250 [14]	55	M	RCC	No data	Current	2	GGO, pleural effusion, atelectasis	565	No data	No data	(+)	Discontinuation, steroid	Death
	Gut and Liver, Vol.4, No. 4, December 2010, 543–546 [22]	74	M	HCC	No data	Former	0.8	Bilateral GGO, interlobular septal thickening	No data	No data	No data	No data	Discontinuation	Recovered
	Clin J Gastroenterol (2012) 5:407–412 [9]	76	M	HCC	No data	No data	0.5	Bilateral GGO	518	No data	No data	(+)	Discontinuation, PSL20mg	Recovered
		75	M	HCC	No data	No data	0.37	Bilateral GGO	1470	43.00%	No data	No data	Discontinuation, HYD1000 mg	Death
		77	M	HCC	3	No data	1.3	Bilateral GGO, interlobular septal thickening	No data	No data	No data	No data	Discontinuation, HYD1000 mg	Death
	Oncol Lett. 2015 Apr;9 (4):1633–1636 [10]	59	M	HCC	No data	Former	5	No data	No data	No data	No data	No data	Discontinuation	Death
Regorafenib	Clin J Gastroenterol 12: 355–360, 2019 [6]	59	M	HCC	0	Former	2	Bilateral GGO	1283	No data	No data	(−)	Discontinuation, steroid	Recovered
	This case	66	M	Rectal cancer	0	Former	3	Bilateral GGO → Bilateral infiltration shadow	578	6.60%	Infiltration of Eosinophils and foamy macrophage into alveolar interstitium	(−)	Discontinuation	Recovered

In conclusion, we report a case of regorafenib‐induced interstitial pneumonia. Japanese Respiratory Society (JRS) guidelines recommend prompt discontinuation of the suspected drug in such cases [23]. While this remains the standard approach for regorafenib‐induced interstitial pneumonia, further case reports are needed to refine management strategies based on individual patient presentations.

## Author Contributions

All authors reviewed and approved the final manuscript.

## Consent

The authors declare that written informed consent was obtained for the publication of this manuscript and accompanying images using the consent form provided by the Journal.

## Conflicts of Interest

The authors declare no conflicts of interest.

## Data Availability

The data that support the findings of this study are available from the corresponding author upon reasonable request.
